# A Case of Delusional Disorder With Abuse of Isoniazid, Rifampicin, Pyrazinamide, and Ethambutol, the First-Line Anti-tuberculosis Therapy Drugs in India

**DOI:** 10.7759/cureus.36893

**Published:** 2023-03-29

**Authors:** Sathiyanarayanan Sathiyamoorthi, Siva Santosh Kumar Pentapati, Sai Sreeja Vullanki, Vijaya Chandra Reddy Avula, Rajeev Aravindakshan

**Affiliations:** 1 Department of Community and Family Medicine, All India Institute of Medical Sciences, Mangalagiri, IND; 2 Department of Psychiatry, All India Institute of Medical Sciences, Mangalagiri, IND

**Keywords:** chatgpt, delusional disorder, abuse, rifampicin, isoniazid, anti-tuberculosis therapy

## Abstract

Tuberculosis (TB) and mental illnesses frequently coexist and are both extremely common worldwide. Through the National Program for Elimination of Tuberculosis (NTEP), anti-tuberculosis therapy (ATT) medications are used to treat tuberculosis in India. We want to report a case 45-year-old patient from the state of Andhra Pradesh, India with comorbid delusional disorder leading to daily ATT drug consumption for the past 20 years. This unusual presentation demonstrates that abuse of a Schedule "H" substance like ATT is also conceivable. To stop "Off-label" purchases, strict measures must be taken. Before beginning ATT, evaluating the patient's mental health may be a wise move.

## Introduction

Globally, both Tuberculosis (TB) and mental illnesses are common and frequently coexist [1]. Reduced treatment-seeking and adherence are linked to poor mental health, which increases morbidity, mortality, transmission, and drug resistance [2]. Additionally, TB can result in neurological symptoms, and some anti-TB drugs have psychiatric side effects like psychosis with Isoniazid and sometimes with Ethambutol [3]. Through the National Program for Elimination of Tuberculosis, anti-tuberculosis therapy (ATT) medications are used to treat tuberculosis in India (NTEP). We want to share an unusual case of comorbid psychotic illness that resulted in daily ATT drug consumption for the past 20 years. This article was previously presented as a conference abstract at the 5th Amrita International Public Health Conference held at Amrita Institute of Medical Sciences, Kochi, India on December 02 - 03, 2022.

## Case presentation

In May 2022, a 45-year-old single man was brought to our tertiary care hospital by his sister with complaints of tremors, sleep disturbances, lack of focus, and tingling and numbness in his lower limbs. The patient was a college graduate from a city in southern India, earned money from rentals, and belonged to a higher socioeconomic class as per BG Prasad Socio-economic Status scale [4]. The patient was not a smoker or alcoholic, as told by the immediate family members. The patient was given the ATT regimen [combination kit of isoniazid (300 mg/day), rifampicin (450 mg/day), ethambutol (800 mg/day), and pyrazinamide (750 mg/day)] for six months because he had pulmonary tuberculosis in 2003. The patient didn't stop taking the medication after 6 months and keeps on getting them through a source at a private nursing home without a prescription, and has been taking it every day for the past 20 years. The patient's family had taken him to numerous hospitals, where numerous doctors had repeatedly advised him that he should stop taking these medications. However, the patient continues to believe that if he does not take the drugs, he will experience cough, indigestion, epigastric discomfort, loose stools, etc. Other common side effects of ATT like orange-coloured urine, blurred vision, etc. were not reported by him.

There was no history of psychiatric disorders in the past or in the family. There was no prior history of head trauma, unconsciousness, or drug use of any kind. The results of the general and systemic examinations were normal. No neurological impairment existed (focal weakness, cranial nerve involvement, or signs of meningeal irritation). A normal blood count, electrolyte levels, liver function, and kidney function were identified by the biochemical analysis. Current fasting blood sugar was 250 mg/dL and HbA1C was 9. These were elevated blood sugar levels. For subsequent visits, radiological investigations such as chest radiographs and neuroimaging were planned. 

A mental health examination done by the psychiatrist revealed poor rapport, anxious affect, impaired judgment, and a lack of insight along with a false, firm, and fixed belief that ATT drugs had maintained his well-being over the years. He was also discovered to have obsessions with seeking out and using these ATT drugs because he thought they would relieve different somatic symptoms. Delusional disorder (ICD 11 -6A24), a harmful pattern of continuous use of non-psychoactive substances (6C4H.11), and diabetes mellitus were the patient's provisional diagnoses (5A11). Delusional disorder, somatic subtype (297.1), and unspecified other substance-related disorders are among the diagnoses taken into account by DSM-5 (292.9). Differential diagnoses for neurotuberculosis, hypochondriacal disorder, drug-induced psychosis, and psychosis not otherwise specified were kept. Atypical antipsychotics were suggested to him, and psychoeducation was carried out. Despite numerous phone calls, follow-up was not possible, which supports the conclusion that the patient lacked insight into the need to stop taking ATT medications as recommended by the treating doctors. The presence of monothematic delusions, the lack of hallucinations or disorderly behaviour, and the absence of pronounced functional impairment are additional characteristics that support the diagnosis of delusional disorder.

## Discussion

This case report likely represents the first to detail a dual diagnosis of psychotic disorder and abuse of drugs that don't cause dependence. In this case study, substance abuse is shown to be secondary to persistent delusional disorder, which is the primary psychiatric diagnosis. The abuse of anti-tubercular drugs with the false belief that one has a disease that can be cured by them was the main focus of this presentation. Higher rates of mental illness are currently prevalent, including psychosis in tuberculosis, according to studies. Psychosis in tuberculosis patients may be brought on by intracranial tuberculoma or may be a side effect of anti-tubercular medications like isoniazid, ciprofloxacin, ethambutol, and rifampicin. Complex causal links exist between tuberculosis and mental illnesses. On the other hand, a tuberculosis diagnosis raises the chance of psychiatric comorbidity. Researchers come to the conclusion that in order to improve tuberculosis cure rates, psychiatric comorbidity must first be recognised and treated [5].

Previous studies show that people with schizophrenia have a higher incidence of TB than controls [6,7]. In one of these studies, the prevalence of schizophrenia was found to be 8.7% in TB patients versus 0.9% in controls (p=0.02) [7]. In a cross-sectional study, patients at an MDR-TB clinic in Ibadan, Nigeria, were examined for more general psychosis. According to this study, patients with MDR-TB had a 33.0% prevalence of psychosis compared to 2.7% of controls. When the prevalence of psychosis in patients with MDR-TB was recalculated to take anti-TB medication-induced psychotic disorders out of the equation, it was 18.3%.

As the patient continued to take ATT drugs and there was no period of abstinence, it was challenging to assess the possibility of drug- (ATT like Isoniazid) induced psychosis. It is typical for antitubercular medications to cause neuropsychiatric adverse drug reactions (ADR). The management of tuberculosis is significantly complicated by psychiatric adverse drug reactions (ADR), which also significantly reduce ATT patients' quality of life. The two anti-TB medications most frequently linked to psychiatric ADR are isoniazid (first-line) and cycloserine (second-line). Ethambutol, ethionamide, and fluoroquinolones have also been linked to neuropsychiatric ADR [8]. We advise clinicians to keep an eye out for psychiatric symptoms throughout the course of treatment as well as at the beginning of ATT.

Because ATT drugs are Schedule "H" substances, their indiscriminate use may result in the emergence of tuberculosis bacillus strains that are resistant to treatment. The abuse of ATT is concerning, particularly in a nation like India where it is simple to obtain these medications over the counter without a prescription. Therefore, strict measures should be implemented to prevent the purchase and use of ATT medications off-label. This underlines the need for integrated programmes that offer care for both mental health and TB, and it implies that prompt interventions that address mental illnesses can stop the abuse of life-saving medications like ATT drugs. Therefore, it is advised that TB and mental health treatments be combined in the WHO End TB Strategy 2015-2035 [9]. In order to deliver holistic care that may prove to be more effective, strict treatment adherence for TB and high-quality mental health support both require significant patient engagement [10].

## Conclusions

This unusual presentation demonstrates that abuse of a Schedule "H" substance like ATT is also conceivable. To stop the sale of ATT medications off-label, strict measures must be taken. Before beginning ATT, evaluating the patient's mental health may be a wise move.

## Figures and Tables

**Figure 1:**
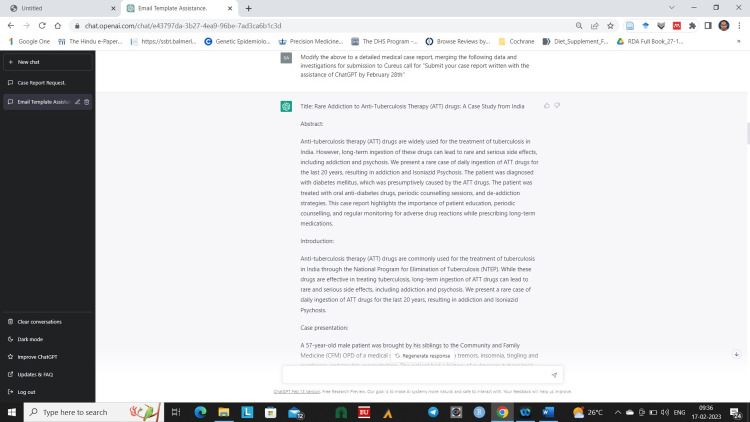
Picture depicting use of ChatGPT while manuscript preparation (1/2)

**Figure 2:**
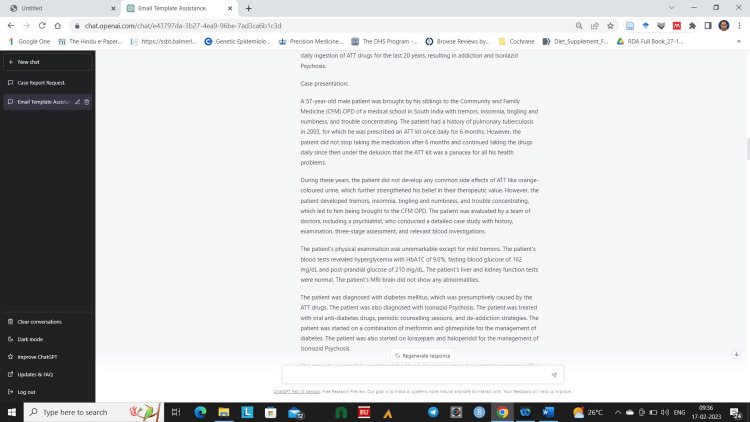
Picture depicting the use of ChatGPT while manuscript preparation (2/2)
